# A Significant Event

**Published:** 1920-03-06

**Authors:** 


### THE PUBLIC WELL-BEING.

### A Significant Event.

Lady Astor's maiden speech in the House of Commons was a novel event in that it was the first time that a woman had addressed that exclusive assembly. Every personal side of the novelty of a woman member of Parliament had been closely explored. Numberless paragraphs had been written about the clothes to be worn, the manner in which the King should address the House in place of the former designation which applied only to men, accommodation, and a hundred other things. More interest seemed to have been displayed in the superficialities than in the fundamentals. But it is a great tribute to Lady Astor herself that her maiden speech delivered in these circumstances of curiosity has, by its force of conviction and honesty, swept aside the smaller stuff and compelled public attention to the bigger significance of women's share in public and legislative work of the country. It is a great ordeal for any new member to address that very critical body of people in the House of Commons. It has many mannerisms and prejudices the non-observance of which may sometimes be disastrous to a newcomer, and Lady Astor's ordeal was intensified because she spoke not only as a new member but as the first woman member. She came through with flying colours, and gave the House a refreshing example of frankness and pluck. She demonstrated what she claimed for women in her second address to the House, two or three days later, that women brought into public life a sort of moral courage which was needed. Superficial dialectics can always command Parliamentary applause, but Lady Astor showed something akin to a passion for social welfare which commanded respect, and her speech showed that her interests in the well-being of the people, in their conditions of life, and their chances of happiness, are not confined to one section only, but are catholic and deeply felt.

The drink question, upon which she addressed the House, is a ticklish subject on which to make a *début*, but she handled it fearlessly, and, in fact, brought into the discussion something of woman's earnestness, which is so valuable. She is clearly bringing to bear upon all questions of public welfare a humane outlook and sympathetic spirit of progress which cannot fail to have the effect of encouraging all women sharing, or contemplating a share in, public life. She has great pioneering opportunities, and she is using them well. Most women will probably have resented the jest of Sir John Rees, who said that he knew Lady Astor had a rod in pickle for him, but that he would be prepared to kiss the rod. Lady Astor's apt reply that he was more than polite might well have stopped at that. Women nowadays expect to be spared these pseudo-gallant pleasantries; they want to be taken seriously and treated seriously, and it would be well for all to recognise and respect that attitude. Lady Astor is not likely to invite the retort given by a Scotsman to the many gay flag-day ladies, that he was keeping his money for the first plain flag-seller he met. She may, however, find it necessary to administer some such similar snubs in a different but analogous set of circumstances.

She has now spoken twice in Parliament, and there are already signs that consideration is being transferred from what she wears to what she contributes to debate. Perhaps, in the initial stages, the sense of novelty has aided Lady Astor in the propagation of her views.

